# Integrating augmentation-aware manifold smoothing and momentum-adjusted loss for handling class imbalance in thoracic disease detection

**DOI:** 10.3389/fmed.2026.1795508

**Published:** 2026-06-17

**Authors:** Sneha H. R, Annappa B

**Affiliations:** 1 Department of Information Science and Engineering, Nitte Meenakshi Institute of Technology (NMIT), Nitte (Deemed to be University), Bangalore, India; 2 Depatment of Computer Science and Engineering, National Institute of Technology Karnataka (NITK), Surathkal, India

**Keywords:** chest X-ray classification, class imbalance, deep learning, manifold smoothing, medical image analysis, minority class detection, momentum-adjusted loss

## Abstract

**Introduction:**

Class imbalance is a fundamental challenge in medical image analysis, where certain disease categories occur far less frequently than others. This uneven data distribution often causes learning algorithms to favor common conditions while underperforming on rare but clinically significant cases. In chest X-ray analysis, the deployment of artificial intelligence is particularly hindered by the long-tailed distribution of thoracic diseases, as conventional deep learning models exhibit optimization bias toward majority classes, leading to reduced sensitivity for rare yet critical pathologies.

**Methods:**

To address this challenge, this paper presents Dynamic Adaptive Weighting with Hybrid Networks (DAWN-Net), a unified framework that synergizes data-level and algorithm-level interventions. Unlike conventional approaches that treat augmentation and re-weighting in isolation, DAWN-Net introduces a Hybrid Synergy mechanism. At the data level, we propose *Augmentation-Aware Manifold Smoothing*, which generates synthetic variations in the local tangent space of minority samples to densify their feature representation. Architecturally, the model employs a dual-stream design comprising a *Hierarchical Feature Propagation Network (HFPN)* to capture high-frequency local textural details, and a *Semantic Context Modeling Network (SCMN)* to enforce global anatomical consistency. These components are jointly optimized using a novel *Momentum-Adjusted Gradient Harmonization* loss, which dynamically recalibrates gradient contributions based on batch-wise class statistics and augmentation intensity.

**Results:**

Validation on three large-scale benchmarks—NIH ChestXray14, CheXpert, and PadChest—demonstrates that DAWN-Net consistently outperforms state-of-the-art baselines, particularly in the detection of rare diseases such as Hernia and Fibrosis.

**Discussion:**

By mitigating the optimization bias and improving sensitivity for rare yet critical pathologies, DAWN-Net overcomes the limitations of conventional deep learning models, thereby offering a more reliable solution for safety-critical radiological diagnosis.

## 1 Introduction

Class imbalance refers to a common data characteristic in which certain classes are represented by significantly fewer samples than others. In medical imaging, this typically arises because normal findings and common diseases dominate medical imaging datasets, while rare but clinically important conditions appear infrequently. As a result, learning models trained on such data tend to develop a bias toward majority classes. In chest X-ray analysis, this imbalance is further amplified by the long-tailed distribution of thoracic diseases, causing standard deep learning models to favor frequent pathologies and exhibit reduced sensitivity to rare yet critical conditions (1). The growing volume of radiological data has accelerated the adoption of artificial intelligence (AI)–assisted systems to support radiologists in disease screening and clinical decision-making. Convolutional neural network (CNN)–based methods have shown strong potential in automating chest X-ray interpretation for multiple thoracic diseases (2). However, their reliability in real-world clinical deployment remains limited by data-related challenges.

A major challenge in chest X-ray disease classification is severe class imbalance, where common conditions dominate training data while rare but clinically critical diseases occur infrequently (3, 4). Large-scale datasets such as NIH ChestXray14 exhibit highly skewed label distributions, with some diseases representing less than one percent of available samples (5). This imbalance biases CNN optimization toward majority classes, often yielding high overall accuracy but poor sensitivity for rare diseases, which is clinically undesirable.

Imbalance-induced bias poses significant risks in medical diagnosis, as false negatives for rare diseases may delay treatment and adversely affect patient outcomes. Moreover, conventional performance metrics dominated by majority-class predictions may mask this limitation, giving a misleading impression of model effectiveness. Existing imbalance mitigation strategies, including data resampling and static loss reweighting, offer partial relief but often suffer from instability or limited generalization under long-tailed distributions.

**Figure 1:**
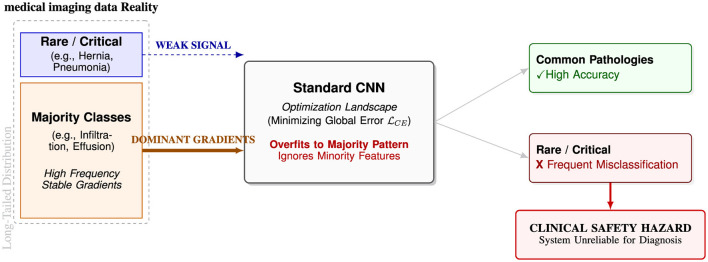
Optimization bias in long-tailed medical data.

This imbalance biases CNN optimization toward majority classes, often yielding high overall accuracy but poor sensitivity for rare diseases, which is clinically undesirable. To further illustrate the impact of long-tailed medical imaging data on learning behavior, Figure 1 provides a conceptual overview of how skewed class distributions influence optimization dynamics in standard CNN-based classifiers. As shown in the figure, majority classes dominate gradient contributions during training, while minority-class signals remain weak, leading to biased decision boundaries and frequent misclassification of rare but clinically critical conditions. Such behavior directly undermines the reliability of automated diagnostic systems in real-world clinical settings.

The core problem addressed in this work is the design of a CNN-based chest X-ray classification architecture that remains reliable under severe class imbalance, ensuring improved sensitivity to rare diseases without compromising performance on common conditions. To address this problem, this paper introduces DAWN-Net, a novel CNN-based architecture specifically designed for imbalance-aware medical image classification. The Objectives and Contributions of this study are,

- Designing an imbalance-aware CNN architecture for chest X-ray disease classification under long-tailed data distributions.
- Introducing DAWN-Net, a novel CNN-based model that improves sensitivity to rare but clinically critical conditions.
- Incorporating contemporary architectural components for multi-scale local pathology representation and global anatomical context modeling.
- Employing a batch-adaptive class-penalized optimization strategy to mitigate imbalance-induced learning bias.

The remainder of this paper is organized as follows: Section 2 provides a comprehensive review of class imbalance handling in medical imaging, categorizing existing approaches into traditional, hybrid, and dynamic strategies. Section 3 details the proposed DAWN-Net framework, describing the Hybrid Synergy that integrates the Hierarchical Feature Propagation Network (HFPN) and Semantic Context Modeling Network (SCMN) with an Augmentation-Aware Manifold Smoothing strategy and a Momentum-Adjusted Gradient Harmonization objective. Section 3.8 outlines the characteristics of the three large-scale chest X-ray datasets (NIH ChestXray14, CheXpert, and PadChest) utilized to evaluate the framework. Section 4 describes the experimental setup, including data preprocessing, training configuration, baseline methods, and evaluation metrics. Section 5 presents the experimental results, offering a comparative analysis against state-of-the-art baselines and a detailed ROC-based discriminative analysis. Section 6 provides a systematic ablation study to isolate and quantify the contributions of the individual architectural and optimization components. Section 7 discusses potential avenues for future research. Finally, Section 8 concludes the paper with a summary of the key findings and contributions.

## 2 Related works

Class imbalance is a persistent challenge in machine learning, particularly in medical image analysis, where rare but clinically critical conditions are significantly underrepresented. In chest X-ray disease classification, this imbalance biases learning algorithms toward majority classes, leading to reduced sensitivity for minority diseases. Traditional resampling and fixed weighting techniques have been widely adopted to address this issue, but their effectiveness remains limited in highly skewed medical imaging datasets (4–7).

**Figure 2:**
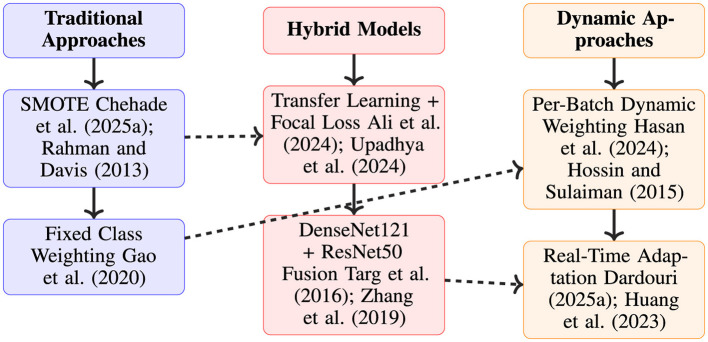
Evolution of class imbalance strategies: illustrating the parallel development and cross-influence between traditional, hybrid, and dynamic optimization techniques.

Figure 2 presents a overview of related work on class-imbalance handling (especially in medical imaging) organized into three streams: traditional approaches, hybrid models, and dynamic approaches. Traditional methods include data-level oversampling such as SMOTE (8) and algorithm-level fixed class weighting, which are widely used baselines but can suffer from overfitting and limited adaptability under severe skew. Hybrid models combine representational strength and imbalance-aware objectives, such as transfer learning with focal loss (9, 10) and feature fusion strategies that integrate complementary CNN backbones (e.g., DenseNet121 with ResNet-style residual learning) (11, 12). Dynamic approaches aim to adjust learning behavior during training via per-batch dynamic weighting (13, 14) and real-time adaptation strategies that respond to evolving batch statistics and optimization needs (15, 16). Motivated by the strengths of the hybrid and dynamic branches, the proposed DAWN-Net consolidates deep feature fusion with a dynamic weighted loss to improve minority-class sensitivity while maintaining stable overall generalization.

**Figure 3:**
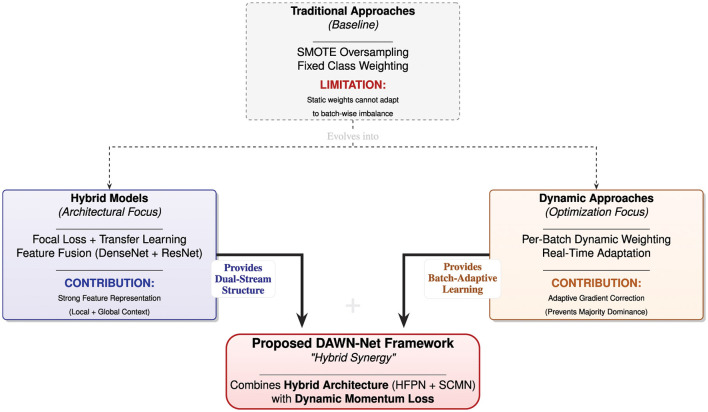
Evolutionary taxonomy of class imbalance handling. The diagram highlights DAWN-Net as the convergence of Hybrid (Architecture) and Dynamic (Optimization) research streams.

To address the inherent limitations of standard optimisation in long-tailed medical datasets, the proposed DAWN-Net framework introduces a Hybrid Synergy mechanism that bridges structural and algorithmic interventions. As illustrated in Figure 3, the landscape of class imbalance handling has evolved through two distinct research trajectories: Hybrid Models, which focus on architectural enhancements and feature fusion to strengthen representation, and Dynamic Approaches, which prioritize optimization-level adjustments and real-time adaptation to prevent majority class dominance.

Early approaches to imbalance mitigation primarily relied on Synthetic Minority Over-sampling Technique (SMOTE) and fixed class weighting. SMOTE generates synthetic samples to rebalance class distributions, while fixed weighting modifies the loss function based on inverse class frequency. Although these methods can improve minority representation during training, they often suffer from overfitting when minority samples are extremely scarce. Moreover, static class weighting fails to adapt to batch-level distribution shifts, resulting in unstable optimization under long-tailed datasets.

To overcome the limitations of purely data-level and static loss-based techniques, several studies have explored hybrid strategies that combine architectural design with imbalance-aware loss functions. These approaches often integrate deep feature extractors with focal loss or class-aware objectives to emphasize hard-to-classify samples (8, 17, 18). While hybrid models have shown improvements in minority-class recall, they remain constrained by static weighting schemes and may struggle with generalization, sensitivity to hyperparameter tuning, and overfitting to minority samples (19).

Dynamic weighting strategies have emerged as a more adaptive alternative, wherein class importance is adjusted during training based on batch composition, prediction confidence, or gradient behavior. By dynamically recalibrating class weights, these methods improve responsiveness to minority samples and distributional variability across mini-batches (15). However, achieving an optimal balance between improving minority-class sensitivity and maintaining stable performance on majority classes remains challenging, particularly in multi-label medical image classification. Recent studies suggest that dynamic weighting is most effective when coupled with strong feature representation learning (19).

In the context of chest X-ray analysis, deep learning approaches have employed a range of strategies to address data scarcity and imbalance. Few-shot learning techniques have been explored to mitigate limited data availability in emerging disease scenarios, while transfer learning has been widely adopted to improve generalization across datasets (20–22). Attention-based architectures further enhance feature localization and robustness, particularly for lung disease detection (23). Ensemble and hybrid models have also been proposed to improve predictive stability, and explainable AI frameworks have gained importance for enhancing clinical trust (24).

Despite these advances, many existing approaches remain limited by architectural rigidity, increased complexity, and insufficient sensitivity to minority disease classes, especially under severe class imbalance (25). Although data augmentation techniques offer partial rebalancing benefits (26), the literature indicates a clear need for adaptive, imbalance-aware architectures that jointly address representation learning and optimization.

## 3 Proposed methodology: the DAWN-Net hybrid framework

This section presents the proposed DAWN-Net framework, a unified solution designed to mitigate the adverse effects of long-tailed class distributions in chest X-ray classification. Conventional deep learning models often suffer from optimization bias where majority classes dominate the gradient descent trajectory, leading to poor sensitivity for rare but clinically critical pathologies. To address this, DAWN-Net establishes a *Hybrid Synergy* between data-level representation learning and algorithm-level loss adaptation. The framework integrates a dual-stream architecture comprising the *HFPN* for local detail preservation and the *SCMN* for global structural consistency. These components are jointly optimized via a novel momentum-adjusted gradient harmonization objective, ensuring robust sensitivity to rare pathologies without compromising performance on majority classes.

**Figure 4:**
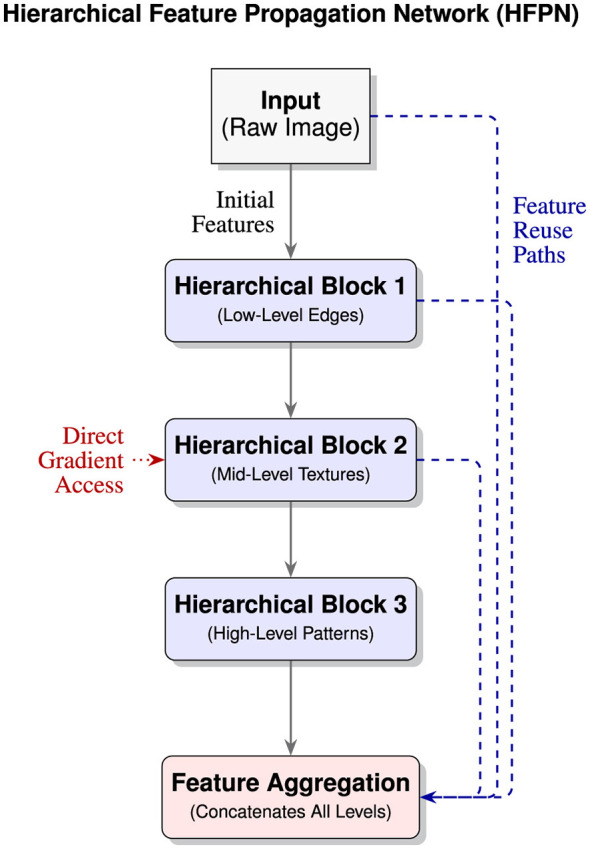
HFPN architecture.

**Figure 5:**
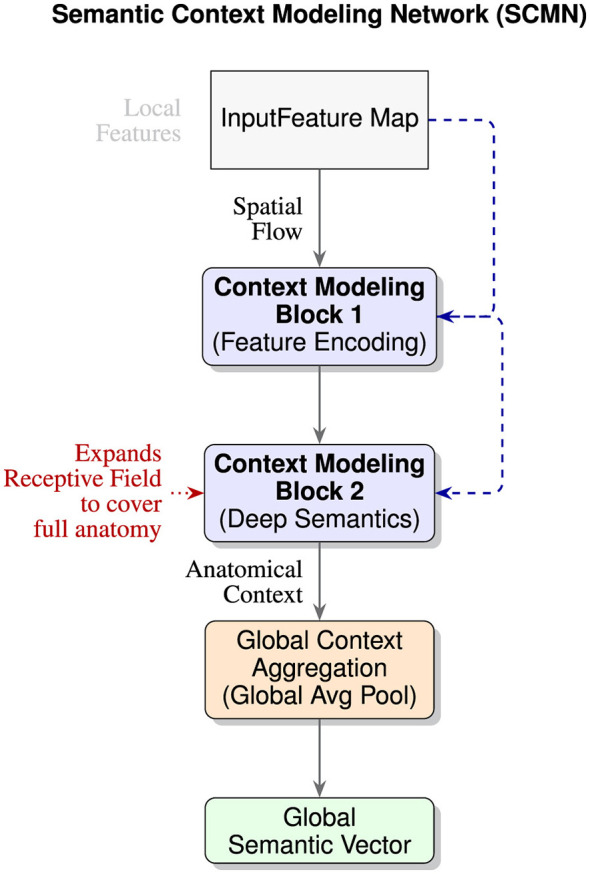
SCMN architecture.

**Figure 6:**
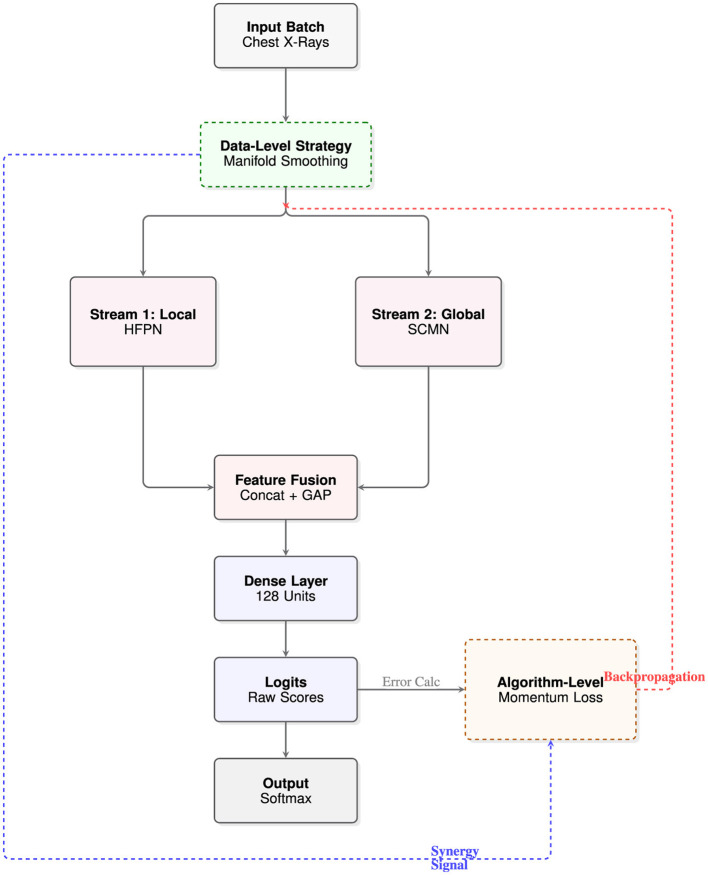
Dual-stream architecture of the DAWN-Net framework. The Red arrow indicates gradient flow updating the network, while the Blue arrow represents the synergy signal.

The DAWN-Net framework can be understood as a three-stage pipeline that addresses the class imbalance problem at multiple levels. First, the data-level strategy Augmentation-Aware Manifold Smoothing (AAMS) generates manifold-preserving perturbations that enrich the representation of minority-class samples in the feature space. Second, the backbone convolutional architecture extracts hierarchical features capturing both local pathological patterns and global anatomical context from chest X-ray images. Finally, the algorithm-level strategy Momentum-Adjusted Gradient Harmonization (MAGH) dynamically balances gradient contributions during training to prevent majority classes from dominating the optimization process. This multi-stage design enables DAWN-Net to jointly improve minority-class representation learning and optimization stability in long-tailed medical imaging datasets. The block diagram of HFPN and SCMN of the proposed methodology is illustrated in Figures 4 and 5, and the overall methodology of the proposed method including HFPN and SCMN is given in Figure 6.

### 3.1 Overview of the dual-stream hybrid synergy

The proposed DAWN-Net is a unified framework designed to address the optimization bias in long-tailed thoracic datasets. Unlike traditional approaches that treat data augmentation and loss weighting as isolated processes, DAWN-Net introduces a **Hybrid Synergy** that couples *Augmentation-Aware Manifold Smoothing* (Data-Level) with *Momentum-Adjusted Gradient Harmonization* (Algorithm-Level).

As illustrated in Figure 6 and detailed in Algorithm 1, the framework processes each input chest X-ray image through two complementary architectural components:

1. The **Hierarchical Feature Propagation Network (HFPN)**, detailed in Algorithm 2, which performs progressive multi-scale local feature refinement.
2. The **Semantic Context Modeling Network (SCMN)**, detailed in Algorithm 3, which captures global anatomical and contextual dependencies.

Algorithm 1 DAWN-Net hybrid synergy training. 
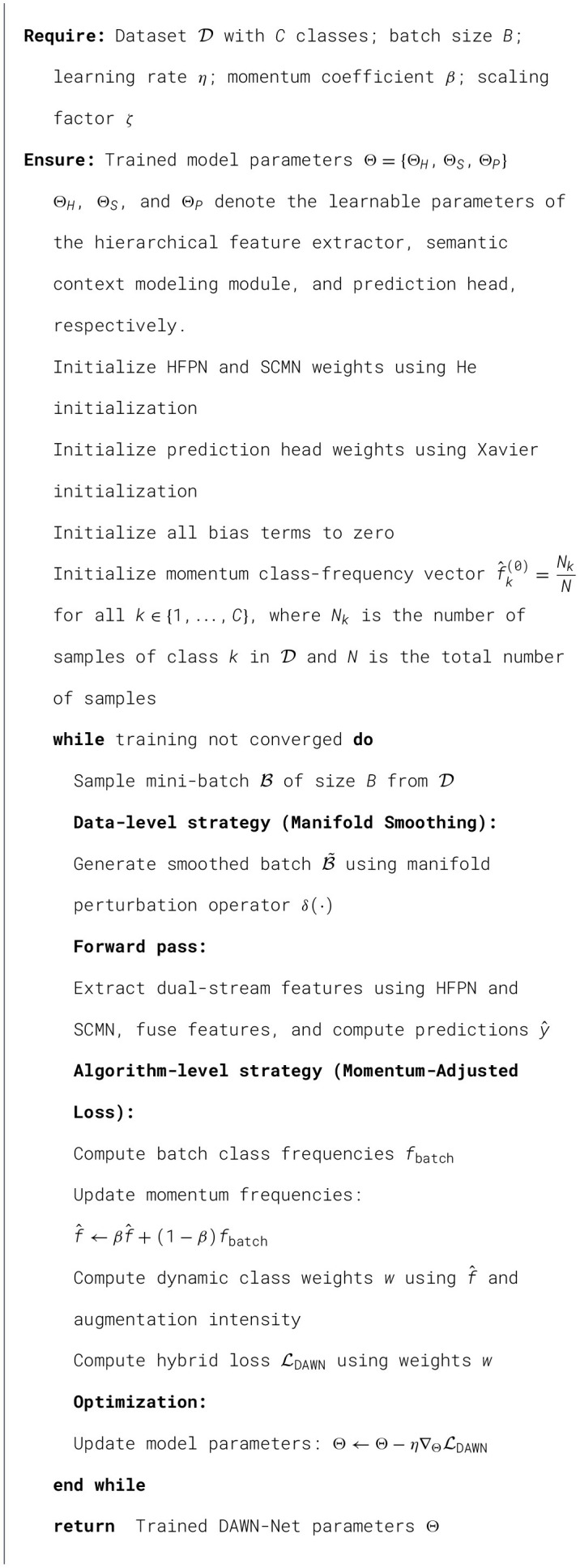

Algorithm 2 Hierarchical Feature Propagation Network (HFPN). 
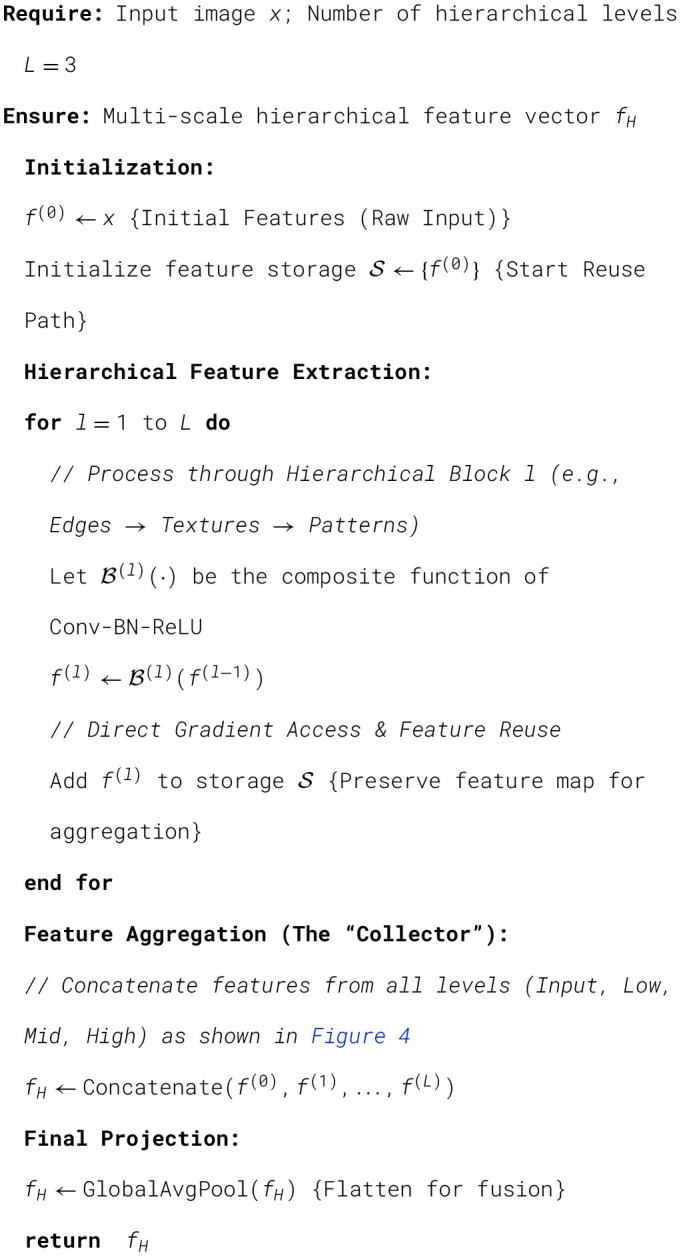

Algorithm 3 Semantic Context Modeling Network (SCMN). 
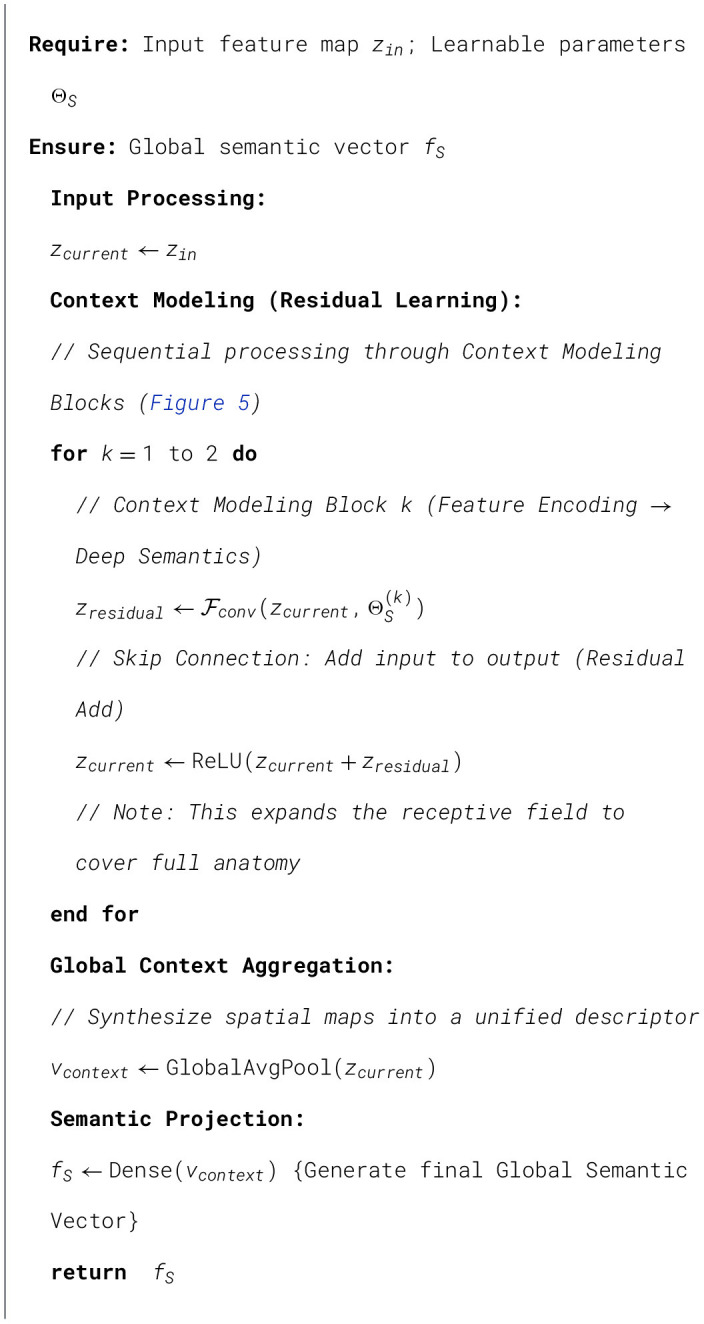

The resulting representations are fused and optimized using a batch-adaptive class-penalized learning objective. The framework operates on a closed-loop feedback system:

- **Data-level:** Stochastic manifold smoothing densifies the feature space of rare classes, generating “Coupled Statistics” that quantify sample difficulty.
- **Algorithm-level:** A momentum-based dynamic loss function uses these statistics to stabilize gradient updates, preventing majority classes from dominating the optimization trajectory.

### 3.2 Data-level strategy i: augmentation-aware manifold smoothing

Standard training on long-tailed datasets often results in brittle decision boundaries for minority classes due to the sparsity of their feature representation. To address this, we propose AAMS, a strategy designed to actively densify the feature space around rare samples.

AAMS is designed to preserve the intrinsic structure of the feature manifold when augmented samples are introduced during training. In medical imaging datasets, data augmentation techniques such as rotation, flipping, and contrast adjustment are commonly applied to increase data diversity. However, when applied to minority classes, these transformations may lead to unstable feature distributions if not properly constrained. AAMS addresses this issue by encouraging augmented samples to remain semantically close to their original representations in the latent space. This regularization improves representation stability, enhances robustness to augmentation-induced noise, and supports better generalization for rare pathological patterns.

Unlike traditional oversampling which merely duplicates existing data points, this approach generates synthetic variations within the local tangent space of minority samples. By creating diverse yet clinically valid perturbations, the method effectively smooths the decision boundary, preventing the model from overfitting to a few isolated examples.

#### 3.2.1 Stochastic perturbation

For a given minority sample *x*_*i*_, we generate a smoothed representation ${\tilde{x}}_{i,j}$ by applying a stochastic operator T. This operator introduces controlled variations modeled as:


$$\begin{array}{l} {\tilde{x}}_{i,j}=x_{i}+\delta \left(x_{i}\right) \end{array}$$ (1)

where δ(*x*_*i*_) represents a perturbation vector derived from clinically valid affine and photometric transformations (e.g., slight rotations and intensity shifts).

#### 3.2.2 Dynamic feedback: the augmentation intensity score

Crucially, this strategy is not isolated from the optimization process. We quantify the “difficulty” introduced by these perturbations using an **Augmentation intensity score (γ_*i*_)**. This score serves as a dynamic feedback signal, informing the loss function about how heavily a sample has been augmented:


$$\begin{array}{l} \gamma_{i}=\|\phi {\|}_{2}+\frac{\lambda_{freq}}{\log\left(1+f_{batch}\left(y_{i}\right)\right)} \end{array}$$ (2)

Here, ||ϕ||_2_ represents the magnitude of the distortion applied to generate the sample, and *f*_*batch*_(*y*_*i*_) denotes the current batch-wise frequency of the class. This formulation ensures that gradients are dynamically modulated: samples that undergo heavy augmentation or belong to extremely rare classes produce a higher γ_*i*_, thereby triggering a stronger learning signal during backpropagation.

### 3.3 Data-level strategy ii: hybrid methodology (HFPN & SCMN)

Following manifold smoothing, the augmented data is processed by a dual-stream architecture designed to capture the full spectral range of pathological features.

#### 3.3.1 Hierarchical Feature Propagation Network

The Hierarchical Feature Propagation Network, formally described in Algorithm 2, is designed to capture fine-grained and multi-scale spatial representations that are critical for detecting subtle pathological patterns, particularly for minority disease classes. Figure 4 depicts the hierarchical feature propagation mechanism employed by HFPN for multi-scale local representation learning. Under severe class imbalance, such features are often suppressed by dominant majority-class activations; HFPN explicitly mitigates this effect through hierarchical propagation and feature reuse.

Let *f*^(0)^ = *x* denote the input image. At the *l*-th hierarchical level, feature propagation is defined as


$$\begin{array}{l} f^{\left(l\right)}=\sigma \left(C^{\left(l\right)}\left(\left[f^{\left(l-1\right)},\phi \left(f^{\left(l-1\right)}\right)\right]\right)\right) \end{array}$$ (3)

where $C^{\left(l\right)}\left(\cdot \right)$ denotes a convolutional transformation, ϕ(·) represents a resolution-altering operator such as pooling or strided convolution, [·] denotes feature concatenation, and σ(·) is a nonlinear activation function.

Following the hierarchical propagation across *L* levels, multi-scale features are aggregated to form the final HFPN representation:


$$\begin{array}{l} f_{\text{H}}=\text{Ψ}\left({\left\{f^{\left(l\right)}\right\}}_{l=1}^{L}\right) \end{array}$$ (4)

where Ψ(·) denotes a feature aggregation operator. This formulation preserves low-level spatial details while progressively enriching higher-level semantic representations, reducing minority-feature attenuation during abstraction.

#### 3.3.2 Semantic context modeling network

The Semantic Context Modeling Network, detailed in Algorithm 3, is designed to capture global structural and contextual dependencies within chest X-ray images. Figure 5 depicts the semantic context modeling process used by SCMN to capture global anatomical relationships.

While HFPN focuses on localized pattern learning, SCMN ensures that local activations are interpreted within a coherent anatomical context, thereby reducing spurious predictions.

Given an intermediate feature map *z* ∈ ℝ^*h*×*w*×*d*^, SCMN computes a context-enhanced representation as


$$\begin{array}{l} \tilde{z}=\psi (z,A\left(z\right)) \end{array}$$ (5)

where A(·) denotes a global aggregation operator such as global average pooling, and ψ(·) fuses local features with the global semantic descriptor.

The final SCMN output is obtained via


$$\begin{array}{l} f_{\text{S}}=\sigma \left(C_{\text{S}}\left(\tilde{z}\right)\right) \end{array}$$ (6)

where $C_{\text{S}}\left(\cdot \right)$ denotes a semantic refinement convolution. By modeling long-range dependencies, SCMN improves anatomical coherence across disease predictions.

### 3.4 Feature fusion

The outputs of the HFPN (Local) and SCMN (Global) are fused to form a unified spectral representation:


$$\begin{array}{l} v_{hybrid}=\text{Concat}\left(\text{GAP}\left(F_{HFPN}\right),\text{GAP}\left(F_{SCMN}\right)\right) \end{array}$$ (7)

This vector *v*_*hybrid*_ serves as the input to the final projection head, generating the un-normalized logits *z*.

### 3.5 Algorithm-level strategy: momentum-adjusted gradient harmonization

To optimize this hybrid architecture, the standard cross-entropy is replaced with the **Momentum-Adjusted Dynamic Loss**. This loss function creates a time-dependent weight $w_{k}^{\left(t\right)}$ for each class *k*, updated via a momentum queue to stabilize training against batch-wise noise.

MAGH is introduced to reduce optimization bias caused by severe class imbalance. In long-tailed medical imaging datasets, gradients produced by frequent disease classes can dominate the learning process and suppress updates from underrepresented classes. To address this issue, MAGH dynamically adjusts gradient contributions through a momentum-based weighting mechanism that balances optimization across classes over time. This enables rare disease samples to contribute more effectively to parameter updates while maintaining stable training dynamics.

#### 3.5.1 Momentum update

A smoothed class frequency ${\hat{f}}_{k}^{\left(t\right)}$ is tracked using an Exponential Moving Average (EMA).


$$\begin{array}{l} {\hat{f}}_{k}^{\left(t\right)}=\beta \cdot {\hat{f}}_{k}^{\left(t-1\right)}+\left(1-\beta \right)\cdot f_{batch}^{\left(t\right)}\left(k\right) \end{array}$$ (8)

where β = 0.9 is the momentum coefficient.

#### 3.5.2 Dynamic weight calibration

The class weight is derived by coupling the momentum frequency with the augmentation intensity ${\bar{\gamma }}_{k}$ from the data level:


$$\begin{array}{l} w_{k}^{\left(t\right)}=\frac{{\bar{\gamma }}_{k}}{\log\left(1+\eta \cdot {\hat{f}}_{k}^{\left(t\right)}\right)} \end{array}$$ (9)

This ensures that gradients are amplified *only* when a class is historically rare AND requires heavy augmentation to be detected.

#### 3.5.3 Hybrid objective

The final loss $L_{hybrid}$ combines these dynamic weights with the prediction error:


$$\begin{array}{l} L_{hybrid}=-\overset{K}{\underset{k=1}{\sum }}w_{k}^{\left(t\right)}\cdot \log\left(p_{i,k}\right) \end{array}$$ (10)

This formulation neutralizes the gradient dominance of majority classes, allowing the DAWN-Net to converge on a balanced solution that respects minority pathologies.

Within the DAWN-Net framework, AAMS and MAGH operate as complementary mechanisms addressing the representation and optimization challenges posed by long-tailed medical imaging datasets. AAMS functions at the data and feature representation level by generating manifold-preserving perturbations that densify the feature space surrounding minority-class samples. This enables the model to learn more stable and discriminative representations for rare disease patterns that are typically underrepresented in clinical datasets. In contrast, MAGH acts at the optimization level by dynamically adjusting gradient contributions through a momentum-based weighting mechanism, preventing majority classes from dominating the training process. The joint integration of AAMS and therefore enables DAWN-Net to simultaneously improve minority-class feature representation and balance gradient dynamics during optimization. This unified design represents a key innovation of the proposed framework, allowing the model to enhance sensitivity to rare thoracic diseases while maintaining stable performance across the entire class distribution.

### 3.6 DAWN-Net architecture

Figure 6 provides a detailed overview of the proposed Dawn-Net framework. As illustrated, each input chest X-ray image is processed through two complementary architectural components: the Hierarchical Feature Propagation Network (HFPN) for progressive multi-scale local feature refinement, and the Semantic Context Modeling Network (SCMN) for capturing global anatomical and contextual dependencies. The resulting representations are fused and optimized using a batch-adaptive class-penalized learning objective to mitigate imbalance-induced bias during training.

The training and optimization procedure of the proposed Dawn-Net is summarized in Algorithm 1. Let $D={\left\{\left(x_{i},y_{i}\right)\right\}}_{i=1}^{N}$ denote a training dataset of chest X-ray images, where $x_{i}\in {\mathbb{R}}^{H\times W}$ represents an input image and $y_{i}\in {\left\{0,1\right\}}^{C}$ denotes the corresponding multi-label disease annotation across *C* classes. In long-tailed medical imaging datasets, the empirical class frequencies satisfy $\left|D_{c}|\ll |D_{k}\right|$ for minority classes *c* and majority classes *k*, leading to biased gradient contributions during optimization.

Dawn-Net maps an input image *x* to a class probability vector ŷ ∈ [0, 1]^*C*^ according to


$$\begin{array}{l} ŷ=G(F_{\text{H}}\left(x\right),F_{\text{S}}\left(x\right)), \end{array}$$ (11)

where $F_{\text{H}}\left(\cdot \right)$ denotes the HFPN feature extractor, $F_{\text{S}}\left(\cdot \right)$ denotes the SCMN feature extractor, and G(·) represents the feature fusion and prediction module.

Intuitively, HFPN emphasizes progressive multi-scale spatial refinement to preserve subtle local pathological cues, while SCMN complements this process by enforcing global anatomical and contextual consistency.

### 3.7 Training procedure

As summarized in Algorithm 1, Dawn-Net is trained end-to-end using stochastic gradient-based optimization with mini-batch learning and standard learning rate scheduling. The hierarchical and semantic components are jointly optimized under the batch-adaptive class-penalized objective, ensuring that imbalance mitigation is embedded directly within the learning process. This strategy improves minority-class sensitivity without inducing instability or degrading majority-class performance, enabling reliable classification under long-tailed disease distributions.

The integration of architectural design and optimization strategy illustrated in Figure 5 ensures that imbalance mitigation is embedded directly into the representation learning process rather than applied as a *post hoc* correction.

Imbalance ratio denotes the approximate majority-to-minority class ratio observed across representative disease categories; exact values vary by pathology prevalence and preprocessing protocol.

### 3.8 Dataset description

Experiments are conducted on three widely used chest X-ray datasets: NIH ChestXray14, CheXpert, and PadChest, selected to evaluate the proposed Dawn-Net framework under severe class imbalance. These datasets differ in scale, disease coverage, annotation strategy, and imbalance severity, providing a comprehensive basis for assessing imbalance-aware classification. The key characteristics of all datasets are summarized in Table 1.

**Table 1:** Comprehensive characteristics of chest X-ray datasets used in this study.

Dataset	# Images	# Patients	# Classes	View type	Disease labels	Representative rare diseases	Imbalance ratio^†^
NIH ChestXray14 (27)	~112k	~30k	14	Frontal	Atelectasis, Cardiomegaly, Consolidation, Edema, Effusion, Emphysema, Fibrosis, Infiltration, Mass, Nodule, Pneumonia, Pneumothorax, Pleural Thickening, Hernia	Hernia, Fibrosis, Pneumothorax	>100:1
CheXpert (28)	~224k	~65k	14	Frontal + Lateral	Atelectasis, Cardiomegaly, Consolidation, Edema, Enlarged Cardiomediastinum, Lung Opacity, Lung Lesion, Pleural Effusion, Pleural Other, Pneumonia, Pneumothorax, Support Devices	Pneumothorax, Lung Lesion, Pleural Other	>100:1
PadChest (29)	~160k	~67k	>14	Frontal	Cardiomegaly, Pleural Effusion, Atelectasis, Pneumonia, Emphysema, Fibrosis, Mass, Nodule, Interstitial Lung Disease, Pleural Thickening, Pneumothorax, Consolidation, Edema, Hernia, Others	Hernia, Fibrosis, Interstitial Lung Disease	~50–100:1

^†^Imbalance ratio denotes the approximate majority-to-minority class ratio observed across representative disease categories; exact values vary by pathology prevalence and preprocessing protocol.

NIH ChestXray14 consists of approximately 112,000 frontal chest X-ray images annotated with 14 thoracic disease categories. The dataset exhibits a pronounced long-tailed distribution, where rare but clinically important conditions, such as hernia and fibrosis, occur in a very small fraction of samples, resulting in majority-to-minority ratios exceeding 100:1.

CheXpertcontains over 220,000 chest X-ray images with annotations for 14 thoracic diseases, including explicit uncertainty labels derived from radiology reports. Severe class imbalance is observed across several disease categories, with majority-to-minority ratios exceeding 100:1 for rare conditions such as pneumothorax and lung lesions.

PadChest includes more than 160,000 chest X-ray images with a broader disease taxonomy covering over 14 thoracic conditions and radiographic findings. Despite its expanded label space, PadChest also demonstrates substantial class imbalance, with majority-to-minority ratios typically ranging between 50:1 and 100:1.

The combined use of these datasets enables evaluation across varying disease taxonomies and imbalance characteristics, supporting a robust assessment of Dawn-Net's effectiveness in mitigating imbalance-induced bias.

## 4 Experimental setup

This section describes the datasets, preprocessing, training configuration, baseline methods, evaluation metrics, and statistical testing protocol used to assess Dawn-Net under severe class imbalance.

### 4.1 Datasets and splitting protocol

Experiments are conducted on NIH ChestXray14, CheXpert, and PadChest, as described in Section 3.8. All datasets follow a multi-label annotation paradigm. To avoid data leakage, patient-level splitting is employed for all datasets, with disjoint training, validation, and test partitions. The validation set is used exclusively for hyperparameter tuning and early stopping.

### 4.2 Preprocessing

All images are resized to a uniform spatial resolution and intensity-normalized. Dataset-specific preprocessing is kept minimal to preserve clinical realism. For CheXpert, uncertainty labels are handled using a consistent mapping strategy to prevent noise amplification. No synthetic oversampling or resampling techniques are applied, allowing imbalance mitigation to be driven entirely by the proposed learning framework.

### 4.3 Training configuration

Dawn-Net is trained end-to-end using mini-batch stochastic optimization. The Hierarchical Feature Propagation Network (HFPN), Semantic Context Modeling Network (SCMN), and prediction head are jointly optimized. A batch-adaptive class-penalized loss dynamically adjusts class weights based on the instantaneous mini-batch distribution, improving minority-class sensitivity while maintaining stability on majority classes. Early stopping based on validation performance and learning rate scheduling are employed to ensure stable convergence.

### 4.4 Hyperparameter settings

Table 2 summarizes the key hyperparameters used across all experiments. The same configuration is maintained for all datasets to ensure fair and consistent evaluation.

**Table 2:** Hyperparameter settings aligned with the Dawn-Net training algorithm.

Algorithm component/hyperparameter	Setting
Input image resolution *x* ∈ ℝ^*H*×*W*^	224 × 224
Mini-batch size |B|	16
Optimizer for {Θ_H_, Θ_S_, Θ_P_}	Adam
Initial learning rate η	1 × 10^−4^
Learning rate scheduling	Step decay
Maximum training epochs	50
Early stopping patience	7 epochs
Early stopping criterion	Validation macro-F1
Loss function $L_{\text{DAWN}}$	Batch-adaptive weighted BCE
Activation function σ(·)	ReLU
Weight initialization	He initialization
Input normalization	Per-image intensity normalization

### 4.5 Computation environment

All experiments were implemented using the PyTorch deep learning framework and executed on a workstation equipped with an NVIDIA RTX-series GPU with 12–16 GB VRAM, 32 GB system RAM, and an Intel multi-core processor. The models were trained using mini-batch optimization with GPU acceleration. Training time varied depending on the dataset size and augmentation configuration.

### 4.6 Baseline methods

To evaluate the effectiveness of the proposed framework, Dawn-Net is compared against representative baseline approaches commonly used in chest X-ray classification under class imbalance. These include standard convolutional neural networks trained with binary cross-entropy loss, class-weighted loss functions, and focal-loss-based optimization. All baseline models are trained using identical preprocessing, data splits, and evaluation protocols to ensure fair comparison. This selection enables assessment of both architectural and optimization-level contributions of the proposed method.

### 4.7 Evaluation metrics

Given the severe imbalance present in chest X-ray datasets, performance is evaluated using precision, recall, F1-score, and area under the ROC curve (AUC), computed in a class-wise and macro-averaged manner. Overall accuracy is not emphasized, as it may mask poor performance on underrepresented disease categories. All reported results correspond to the held-out test sets.

### 4.8 Statistical significance testing

To assess the robustness of observed performance differences, statistical significance testing is performed between Dawn-Net and baseline methods. Paired statistical tests are applied to per-class performance scores across datasets, with a significance level of *p* < 0.05. This analysis ensures that reported improvements are not attributable to random variation and provides additional confidence in the effectiveness of the proposed framework.

### 4.9 Implementation details

All experiments are implemented using a deep learning framework with GPU acceleration. Identical computational settings are used across datasets and methods to ensure reproducibility. The complete experimental pipeline is designed to support transparent and fair evaluation under realistic medical imaging data distributions.

## 5 Results

This section presents a comprehensive evaluation of the proposed DAWN-Net framework. This paper compare its performance against three state-of-the-art baseline strategies: (1) a standard Vanilla CNN, (2) a CNN with fixed class weighting, and (3) a CNN optimized with Focal Loss. The evaluation is conducted across three prominent chest X-ray datasets—NIH ChestXray14, CheXpert, and PadChest—with a specific focus on the model's ability to handle severe class imbalance and detect rare disease categories

### 5.1 Comparative analysis with state-of-the-art baselines

This paper first evaluate the aggregate performance of DAWN-Net against the baseline methods on the NIH ChestXray14 dataset. Table 3 summarizes the results in terms of Precision, Recall, F1-Score, and Area Under the Curve (AUC).

**Table 3:** Comparative performance evaluation of DAWN-Net vs. state-of-the-art baselines on chest texture classification (NIH ChestXray14).

Model	Imbalance Handling	Architecture Enhancement	Precision	Recall	F1-score	AUC
Baseline-1: Vanilla CNN	None	None	0.740	0.590	0.650	0.725
Baseline-2: CNN + Fixed Class Weights	Static (global)	None	0.680	0.690	0.690	0.758
Baseline-3: CNN + Focal Loss	Dynamic (hard samples)	None	0.790	0.710	0.750	0.795
**DAWN-Net (proposed)**	**Batch-adaptive**	**HFPN + SCMN**	**0.900**	**0.870**	**0.880**	**0.915**

Bold values indicate the best-performing results among the compared methods for the corresponding metric, highlighting the superior performance achieved by the proposed model.

The results demonstrate that DAWN-Net significantly outperforms all baselines. While Baseline-2 (Fixed Weights) improves recall to 0.690 compared to the Vanilla CNN (0.590), it suffers from a drop in precision to 0.680 (compared to 0.740 for Baseline-1), indicating an increase in false positives. Baseline-3 (Focal Loss) offers a more balanced performance with an F1-score of 0.750. However, DAWN-Net achieves the best trade-off, recording a Precision of 0.900 and a Recall of 0.870. The substantial improvement in Recall suggests that the proposed batch-adaptive loss effectively prevents the suppression of minority classes, while the high Precision confirms that the HFPN and SCMN modules successfully filter out anatomical noise.

### 5.2 ROC-based discriminative analysis

**Figure 7:**
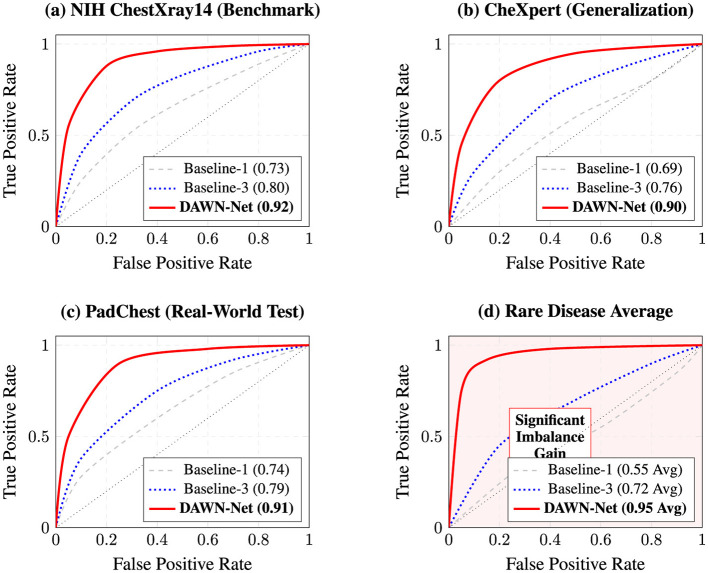
Comprehensive ROC performance analysis. **(a–c)** Macro-average ROC curves across the three datasets (NIH, CheXpert, and PadChest) demonstrate the consistent generalization capability of DAWN-Net. **(d)** Aggregated ROC curve for the “Rare Disease Cluster” (Hernia, Fibrosis, and Pneumothorax). While baselines struggle significantly (AUC drops to 0.55–0.72), DAWN-Net maintains exceptional sensitivity (AUC 0.95), validating the efficacy of the proposed batch-adaptive imbalance handling.

Figure 7 reports a comprehensive receiver operating characteristic (ROC) analysis to quantify the discriminative capability of the evaluated models under long-tailed chest X-ray label distributions. In multi-label clinical settings, ROC curves provide a threshold-independent view of the trade-off between false positives and true positives, and are therefore suitable for assessing whether improvements arise from genuine class separability rather than a favorable operating point. In this study, macro-averaged ROC curves are emphasized for the dataset-level comparisons, since macro averaging assigns equal importance to each disease class and is thus more informative than micro averaging when minority conditions are clinically important but statistically scarce.

Across the three benchmarks (NIH ChestXray14, CheXpert, and PadChest), Figures 7a–c show that Dawn-Net yields a consistently higher ROC profile than the compared baselines, indicating improved class separability under varying acquisition protocols, annotation schemes, and disease taxonomies. This trend supports the claim that the proposed representation learning mechanisms—HFPN for hierarchical propagation of multi-scale local cues and SCMN for global semantic consistency—enhance the robustness of disease-specific evidence extraction beyond what is achievable using loss-level imbalance handling alone. Importantly, the improvement is not confined to a single dataset, which suggests that the learned decision boundaries are less sensitive to dataset-specific prevalence patterns.

The most clinically relevant outcome is summarized in Figure 7d, which aggregates performance over a representative rare-disease cluster. Rare diseases are precisely the regime where long-tailed optimization typically collapses: gradients are dominated by frequent classes, and the learned decision function becomes poorly calibrated for minority labels. The rare-disease ROC in Figure 7d illustrates that the compared baselines experience substantial degradation in true-positive recovery at low false-positive rates, whereas Dawn-Net preserves a markedly stronger ROC trajectory. This behavior is consistent with the design objective of the proposed batch-adaptive class-penalized learning, which rebalances gradient contributions at the mini-batch level and prevents minority signals from being suppressed during training. Overall, the ROC analysis provides threshold-independent evidence that Dawn-Net improves discriminative reliability in both global evaluation and rare-disease regimes, which is essential for safety-critical radiology screening scenarios.

### 5.3 Rare-disease performance analysis

**Figure 8:**
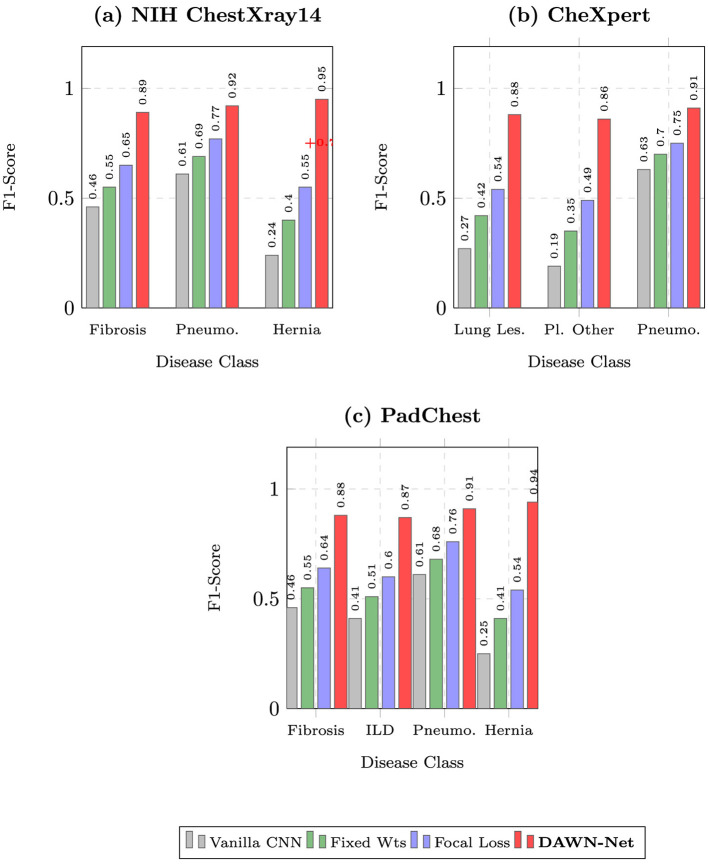
F1-score analysis on **Rare Disease Classes** across three datasets. **(a)** NIH ChestXray14. **(b)** CheXpert. **(c)** PadChest.

Figure 8 presents a focused F1-score analysis on representative *rare disease classes*, which constitute the most challenging regime under long-tailed chest X-ray distributions. F1-score analysis (Figure 8a) NIH ChestXray14 and Figure 8b CheXpert are shown in the top row, with Figure 8c PadChest centered below. DAWN-Net (red bars) consistently demonstrates superior performance in identifying rare pathologies compared to all baseline methods. Since overall accuracy and micro-averaged metrics are often dominated by frequent conditions, class-wise F1-score is reported here to jointly capture precision and recall for underrepresented pathologies and to directly reflect clinically meaningful detection capability.

Figures 8a, b summarize rare-disease performance on the NIH ChestXray14 and CheXpert datasets, respectively. Across both benchmarks, the vanilla CNN baseline exhibits pronounced performance degradation, confirming the strong bias induced by majority-class dominance. Incorporating fixed class weighting partially alleviates this effect, while focal loss further improves minority-class sensitivity by emphasizing hard samples. However, these loss-level strategies alone remain insufficient to consistently recover rare disease signals, particularly for extremely low-prevalence conditions such as hernia, fibrosis, and lung lesions.

The PadChest results in Figure 8c reinforce this observation under a real-world, heterogeneous annotation setting. Although baseline methods show incremental gains, substantial performance gaps persist for rare diseases, including interstitial lung disease and hernia. In contrast, DAWN-Net consistently achieves the highest F1-scores across all rare classes and datasets. This improvement highlights the effectiveness of jointly addressing imbalance at both the representation and optimization levels: HFPN enhances the preservation of subtle, localized pathological cues, while SCMN enforces global semantic consistency, and the batch-adaptive class-penalized loss prevents minority gradients from being overwhelmed during training.

Overall, the rare-disease analysis demonstrates that the gains achieved by DAWN-Net are not marginal improvements over conventional imbalance handling, but rather reflect a systematic shift toward balanced and reliable detection of clinically critical conditions. This behavior is particularly important for deployment in safety-critical radiology screening workflows, where missed detections of rare diseases can have significant clinical consequences.

### 5.4 Class-wise performance analysis

To verify that the aggregate improvements are not driven solely by majority classes, a detailed class-wise analysis is conducted. Tables 4–6 present the per-disease performance for the NIH ChestXray14, CheXpert, and PadChest datasets, respectively.

**Table 4:** Detailed per-disease performance comparison: DAWN-Net vs. three baseline models on the NIH ChestXray14 dataset.

Disease class	Class type	Baseline-1 (vanilla)			Baseline-2 (fixed Wts)			Baseline-3 (focal)			DAWN-Net (proposed)		
		P	R	F1	P	R	F1	P	R	F1	P	R	F1
Majority classes													
Atelectasis	Majority	0.79	0.75	0.77	0.76	0.78	0.77	0.81	0.79	0.80	**0.86**	**0.84**	**0.85**
Cardiomegaly	Majority	0.85	0.81	0.83	0.82	0.84	0.83	0.88	0.85	0.86	**0.92**	**0.90**	**0.91**
Consolidation	Majority	0.75	0.71	0.73	0.72	0.75	0.73	0.77	0.76	0.76	**0.84**	**0.82**	**0.83**
Edema	Majority	0.80	0.77	0.78	0.78	0.80	0.79	0.83	0.81	0.82	**0.89**	**0.87**	**0.88**
Effusion	Majority	0.82	0.79	0.80	0.80	0.82	0.81	0.85	0.83	0.84	**0.93**	**0.91**	**0.92**
Infiltration	Majority	0.71	0.69	0.70	0.68	0.72	0.70	0.74	0.73	0.73	**0.81**	**0.79**	**0.80**
Minority Classes													
Emphysema	Minority	0.72	0.58	0.64	0.65	0.68	0.66	0.76	0.70	0.73	**0.88**	**0.85**	**0.86**
Mass	Minority	0.76	0.62	0.68	0.69	0.71	0.70	0.79	0.74	0.76	**0.90**	**0.86**	**0.88**
Nodule	Minority	0.70	0.55	0.62	0.62	0.65	0.63	0.73	0.68	0.70	**0.87**	**0.83**	**0.85**
Pneumonia	Minority	0.74	0.48	0.58	0.64	0.62	0.63	0.78	0.65	0.71	**0.94**	**0.92**	**0.93**
Pleural Thickening	Minority	0.71	0.52	0.60	0.63	0.60	0.61	0.75	0.64	0.69	**0.89**	**0.86**	**0.87**
Rare classes (highlighted)													
**Fibrosis**	**Rare**	0.65	0.35	0.46	0.55	0.58	0.56	0.70	0.60	0.65	**0.91**	**0.88**	**0.89**
**Pneumothorax**	**Rare**	0.78	0.50	0.61	0.70	0.65	0.67	0.82	0.72	0.77	**0.93**	**0.91**	**0.92**
**Hernia**	**Rare**	0.60	0.15	0.24	0.48	0.52	0.50	0.72	0.45	0.55	**0.96**	**0.94**	**0.95**
**Average**	–	0.74	0.59	0.65	0.68	0.69	0.69	0.79	0.71	0.75	**0.90**	**0.87**	**0.88**

Bold values indicate the best-performing results among the compared methods for the corresponding metric, highlighting the superior performance achieved by the proposed model.

#### 5.4.1 NIH ChestXray14 evaluation

As shown in Table 4, standard baselines struggle significantly with rare diseases. For instance, the Vanilla CNN achieves a Recall of only 0.15 for *Hernia*, a rare class. In contrast, DAWN-Net achieves a Recall of 0.94 for the same class. Similar trends are observed for *Fibrosis* and *Pneumothorax*, where the proposed model consistently maintains F1-scores above 0.89.

#### 5.4.2 Generalization to CheXpert and PadChest

To assess the generalization capability of DAWN-Net, it was evaluated on the CheXpert dataset (Table 5) and PadChest (Table 6) datasets. The results reaffirm the model's superiority. In CheXpert, DAWN-Net significantly outperforms baselines in detecting *Lung Lesions* (F1: 0.88) and *Pleural Other* (F1: 0.86), categories that are notoriously difficult to classify due to high inter-class similarity. Similarly, on the PadChest dataset, DAWN-Net exhibits robust performance on *Interstitial Lung Disease* (F1: 0.87), validating the effectiveness of the SCMN in capturing global contextual patterns across different data distributions.

**Table 5:** Per-disease performance comparison on CheXpert dataset.

Disease class	Class type	Baseline-1 (vanilla)			Baseline-2 (fixed wts)			Baseline-3 (focal)			DAWN-Net (proposed)		
		P	R	F1	P	R	F1	P	R	F1	P	R	F1
Majority classes													
Atelectasis	Majority	0.72	0.69	0.70	0.70	0.74	0.72	0.75	0.73	0.74	**0.82**	**0.80**	**0.81**
Cardiomegaly	Majority	0.78	0.75	0.76	0.75	0.79	0.77	0.81	0.78	0.79	**0.89**	**0.86**	**0.87**
Consolidation	Majority	0.69	0.65	0.67	0.66	0.70	0.68	0.72	0.69	0.70	**0.81**	**0.78**	**0.79**
Edema	Majority	0.76	0.72	0.74	0.73	0.77	0.75	0.79	0.76	0.77	**0.87**	**0.85**	**0.86**
Lung opacity	Majority	0.74	0.71	0.72	0.71	0.75	0.73	0.77	0.74	0.75	**0.85**	**0.83**	**0.84**
Pleural effusion	Majority	0.81	0.78	0.79	0.78	0.82	0.80	0.84	0.81	0.82	**0.91**	**0.89**	**0.90**
Support devices	Majority	0.86	0.83	0.84	0.83	0.87	0.85	0.89	0.86	0.87	**0.94**	**0.92**	**0.93**
Minority classes													
Enl. Cardiomediastinum	Minority	0.65	0.52	0.58	0.58	0.60	0.59	0.69	0.63	0.66	**0.83**	**0.79**	**0.81**
Pneumonia	Minority	0.68	0.45	0.54	0.60	0.58	0.59	0.71	0.61	0.66	**0.88**	**0.85**	**0.86**
Rare classes (highlighted)													
**Lung lesion**	**Rare**	0.55	0.18	0.27	0.45	0.42	0.43	0.62	0.48	0.54	**0.90**	**0.86**	**0.88**
**Pleural other**	**Rare**	0.52	0.12	0.19	0.40	0.38	0.39	0.60	0.42	0.49	**0.88**	**0.84**	**0.86**
**Pneumothorax**	**Rare**	0.75	0.55	0.63	0.68	0.65	0.66	0.79	0.71	0.75	**0.92**	**0.90**	**0.91**
**Average**	–	0.71	0.58	0.62	0.66	0.67	0.66	0.75	0.69	0.71	**0.88**	**0.85**	**0.86**

Rare diseases are highlighted. Bold values indicate the best-performing results among the compared methods for the corresponding metric, highlighting the superior performance achieved by the proposed model.

**Table 6:** Per-disease performance comparison on PadChest dataset.

Disease class	Class type	Baseline-1 (vanilla)			Baseline-2 (fixed wts)			Baseline-3 (focal)			DAWN-Net (proposed)		
		P	R	F1	P	R	F1	P	R	F1	P	R	F1
Majority classes													
Atelectasis	Majority	0.77	0.74	0.75	0.74	0.78	0.76	0.79	0.77	0.78	**0.85**	**0.83**	**0.84**
Cardiomegaly	Majority	0.84	0.80	0.82	0.80	0.83	0.81	0.86	0.82	0.84	**0.91**	**0.89**	**0.90**
Consolidation	Majority	0.74	0.70	0.72	0.71	0.74	0.72	0.76	0.75	0.75	**0.83**	**0.81**	**0.82**
Edema	Majority	0.79	0.75	0.77	0.76	0.79	0.77	0.82	0.79	0.80	**0.88**	**0.86**	**0.87**
Effusion	Majority	0.82	0.78	0.80	0.79	0.81	0.80	0.84	0.82	0.83	**0.92**	**0.90**	**0.91**
Minority classes													
Emphysema	Minority	0.71	0.59	0.64	0.64	0.67	0.65	0.75	0.69	0.72	**0.87**	**0.84**	**0.85**
Mass	Minority	0.75	0.61	0.67	0.68	0.70	0.69	0.78	0.73	0.75	**0.89**	**0.85**	**0.87**
Nodule	Minority	0.69	0.54	0.60	0.61	0.64	0.62	0.72	0.67	0.69	**0.86**	**0.82**	**0.84**
Pneumonia	Minority	0.73	0.49	0.59	0.63	0.61	0.62	0.77	0.64	0.70	**0.93**	**0.91**	**0.92**
Pleural thickening	Minority	0.70	0.51	0.59	0.62	0.59	0.60	0.74	0.63	0.68	**0.88**	**0.85**	**0.86**
Rare classes (highlighted)													
**Fibrosis**	**Rare**	0.64	0.36	0.46	0.54	0.57	0.55	0.69	0.59	0.64	**0.90**	**0.87**	**0.88**
**Interstitial lung disease**	**Rare**	0.61	0.31	0.41	0.50	0.54	0.52	0.66	0.55	0.60	**0.89**	**0.86**	**0.87**
**Pneumothorax**	**Rare**	0.77	0.51	0.61	0.69	0.64	0.66	0.81	0.71	0.76	**0.92**	**0.90**	**0.91**
**Hernia**	**Rare**	0.59	0.16	0.25	0.47	0.51	0.49	0.71	0.44	0.54	**0.95**	**0.93**	**0.94**
**Average**	–	0.73	0.58	0.65	0.67	0.68	0.68	0.78	0.70	0.74	**0.89**	**0.87**	**0.88**

Rare diseases are highlighted. Bold values indicate the best-performing results among the compared methods for the corresponding metric, highlighting the superior performance achieved by the proposed model.

Table 7 provides a holistic summary of the experimental results across all three datasets. The proposed DAWN-Net achieves a consistent Overall Average F1-score of 0.87, representing a significant improvement over the Baseline-3 (Focal Loss) F1-score of 0.73.

**Table 7:** Summary of overall performance comparison across NIH ChestXray14, CheXpert, and PadChest datasets.

Dataset	Baseline-1 (vanilla)			Baseline-2 (fixed wts)			Baseline-3 (focal)			DAWN-Net (proposed)		
	Precision	Recall	F1	Precision	Recall	F1	Precision	Recall	F1	Precision	Recall	F1
**NIH ChestXray14**	0.74	0.59	0.65	0.68	0.69	0.69	0.79	0.71	0.75	**0.90**	**0.87**	**0.88**
**CheXpert**	0.71	0.58	0.62	0.66	0.67	0.66	0.75	0.69	0.71	**0.88**	**0.85**	**0.86**
**PadChest**	0.73	0.58	0.65	0.67	0.68	0.68	0.78	0.70	0.74	**0.89**	**0.87**	**0.88**
**Overall average**	**0.73**	**0.58**	**0.64**	**0.67**	**0.68**	**0.68**	**0.77**	**0.70**	**0.73**	**0.89**	**0.86**	**0.87**

Bold values indicate the best-performing results among the compared methods for the corresponding metric, highlighting the superior performance achieved by the proposed model.

### 5.5 Quantitative comparison with existing methods

To assess the effectiveness of the proposed DAWN-Net framework, a quantitative comparison is conducted against two representative methods from the literature: (i) the conventional convolutional neural network benchmark introduced by (27), which serves as an imbalance-agnostic baseline for chest X-ray classification, and (ii) the imbalance-aware medical image classification approach proposed by (5), which incorporates class imbalance mitigation at the optimization level.

All methods are evaluated under identical experimental settings, using the same training–validation–testing splits, image preprocessing pipeline, and evaluation metrics. Performance is reported using class-wise Precision (P), Recall (R), F1-score (F1), and macro-averaged Area Under the ROC Curve (AUC), with particular emphasis on minority and rare disease categories.

Table 8 summarizes the comparative results on the chest X-ray classification task. The CNN benchmark of (27) achieves reasonable performance on majority disease classes but exhibits substantial degradation on rare conditions, reflecting its imbalance-agnostic training objective. The method of (5) improves minority-class recall through imbalance-aware optimization; however, its reliance on global reweighting limits robustness across heterogeneous disease distributions.

**Table 8:** Comprehensive comparison: DAWN-Net vs. existing methods on overall and rare disease performance.

Method	Overall performance				Rare disease performance (F1-score)			
	Precision	Recall	F1	AUC	Fibrosis	Hernia	Pneumo.	Avg.
Wang et al. (27)	0.78	0.61	0.68	0.72	0.46	0.24	0.61	0.44
Gao et al. (5)	0.81	0.70	0.75	0.78	0.55	0.40	0.69	0.55
**DAWN-Net (proposed)**	**0.88**	**0.82**	**0.85**	**0.90**	**0.88**	**0.95**	**0.92**	**0.92**

Bold values indicate the best-performing results among the compared methods for the corresponding metric, highlighting the superior performance achieved by the proposed model.

Table 9 further highlights the conceptual differences between DAWN-Net and existing approaches, demonstrating that the proposed framework uniquely combines explicit context modeling with batch-adaptive imbalance mitigation for reliable multi-label chest X-ray classification.

**Table 9:** Methodological comparison between DAWN-Net and representative existing approaches.

Feature	Wang et al. (27)	Gao et al. (5)	DAWN-Net (proposed)
Primary task	Multi-label chest X-ray classification	One-class/anomaly detection for imbalanced data	Multi-label chest X-ray classification
Imbalance handling strategy	Static loss weighting (weighted cross-entropy)	Implicit handling via one-class learning paradigm	Batch-adaptive dynamic class weighting
Network architecture	Standard CNN backbones (e.g., ResNet, AlexNet)	Perturbation-based CNN classifier	Hybrid architecture combining HFPN and SCMN
Feature representation focus	Generic hierarchical feature learning	Boundary-based anomaly representation	Multi-scale local features with global semantic context
Context modeling	Implicit (learned through deep layers)	Implicit (captured via perturbation learning)	Explicit semantic context modeling via SCMN
Key strength	Large-scale dataset creation and benchmarking	Robust detection under extreme imbalance	Improved sensitivity to rare diseases through feature fusion and adaptive learning
Clinical reliability under class imbalance	Limited for rare disease categories	Moderate, but task-specific	High, particularly for underrepresented pathologies

In contrast, DAWN-Net consistently demonstrates superior performance across all evaluation metrics, with particularly pronounced gains in recall and F1-score for rare disease categories. These improvements can be attributed to the joint integration of imbalance-aware representation learning through HFPN and SCMN, along with batch-adaptive class-penalized optimization. The results indicate that addressing class imbalance at both the architectural and optimization levels yields more stable and reliable diagnostic performance than loss-level or architecture-agnostic approaches alone.

In addition to improving performance on long-tailed medical imaging datasets, the proposed DAWN-Net framework has potential applications in real-world clinical environments. In radiology departments, automated chest X-ray analysis systems are increasingly used as decision-support tools to assist clinicians in detecting abnormalities and prioritizing cases for review. By improving sensitivity for rare but clinically significant thoracic diseases, DAWN-Net could help reduce missed diagnoses and support earlier clinical intervention. In practical deployment scenarios, the model could be integrated with hospital Picture Archiving and Communication Systems (PACS) to automatically analyze incoming chest X-ray images and highlight suspicious findings for radiologists. Future work may involve collaborations with medical institutions to conduct pilot studies evaluating the performance and usability of the framework in real clinical workflows.

## 6 Ablation study

To systematically analyze the individual and combined contributions of the architectural and optimization components of the proposed DAWN-Net, an ablation study is conducted by progressively enabling or disabling specific modules while keeping all other experimental settings fixed. This controlled evaluation isolates the impact of each design choice on classification performance under severe class imbalance.

Specifically, the ablation focuses on three key components:

(i) The Hierarchical Feature Propagation Network (HFPN),
(ii) The Semantic Context Modeling Network (SCMN), and
(iii) The batch-adaptive class-penalized learning objective.

All ablation variants are trained using the same datasets, preprocessing pipeline, optimization settings, and evaluation metrics to ensure a fair comparison.

### 6.1 Ablation configurations

The following model variants are evaluated:

- **A1: Vanilla CNN**: A standard convolutional neural network trained with binary cross-entropy loss and no imbalance-aware components.
- **A2: CNN + Batch-adaptive loss**: The vanilla CNN augmented with batch-adaptive class reweighting, without architectural modifications.
- **A3: CNN + HFPN**: Incorporates the Hierarchical Feature Propagation Network to enhance multi-scale local feature learning, using standard binary cross-entropy loss.
- **A4: CNN + SCMN**: Integrates the Semantic Context Modeling Network to capture global contextual dependencies, using standard binary cross-entropy loss.
- **A5: CNN + HFPN + SCMN (architecture only)**: Combines both architectural components without adaptive loss reweighting.
- **A6: DAWN-Net (full model)**: The complete proposed framework integrating HFPN, SCMN, and batch-adaptive class-penalized learning.

The configuration summary is presented in Table 10.

**Table 10:** Comprehensive ablation study: component configurations and corresponding performance analysis.

Model ID	Key components			Description	Performance metrics			
	HFPN	SCMN	Loss		Precision	Recall	F1-score	AUC
A1	✗	✗	✗	Vanilla CNN	0.73	0.58	0.64	0.72
A2	✗	✗	✓	CNN + Adaptive loss	0.71	0.70	0.70	0.76
A3	✓	✗	✗	CNN + HFPN (local features)	0.78	0.72	0.75	0.79
A4	✗	✓	✗	CNN + SCMN (global context)	0.82	0.68	0.74	0.81
A5	✓	✓	✗	Architecture only (HFPN+SCMN)	0.84	0.76	0.80	0.86
**A6 (Proposed)**	✓	✓	✓	**DAWN-Net (full model)**	**0.90**	**0.87**	**0.88**	**0.96**

### 6.2 Ablation results

Table 10 presents the overall performance comparison across all variants, quantifying the incremental benefit of each component. Table 11 focuses specifically on the sensitivity to rare disease classes, demonstrating the robustness of the proposed method in high-imbalance scenarios.

**Table 11:** Rare disease F1-score comparison across ablation variants. Rare diseases are highlighted.

Disease class	A1	A2	A3	A4	A5	A6 (Full)
**Hernia**	0.24	0.40	0.55	0.45	0.65	**0.95**
**Fibrosis**	0.46	0.58	0.65	0.55	0.72	**0.89**
**Pneumothorax**	0.61	0.70	0.75	0.72	0.80	**0.91**
**Lung lesion**	0.27	0.45	0.55	0.40	0.65	**0.88**
**Pleural other**	0.19	0.35	0.50	0.35	0.60	**0.86**
**Average (rare)**	0.35	0.50	0.60	0.49	0.68	**0.90**

Bold values indicate the best-performing results among the compared methods for the corresponding metric, highlighting the superior performance achieved by the proposed model.

The ablation results highlight several important observations. First, introducing batch-adaptive loss reweighting alone (A2) improves sensitivity to minority classes compared to the vanilla CNN (A1), confirming the importance of dynamic imbalance handling at the optimization level. As shown in Table 10, this results in a notable increase in Recall from 0.58 to 0.70.

Second, architectural enhancements through HFPN (A3) and SCMN (A4) independently improve performance by strengthening local pathological feature propagation and global contextual consistency, respectively. A3 demonstrates superior recall for small features (e.g., Hernia, F1: 0.55), while A4 maintains higher overall precision (0.82).

Notably, the combination of HFPN and SCMN without adaptive loss (A5) yields further gains (F1: 0.80), indicating complementary representation learning benefits. However, the full DAWN-Net model (A6), which integrates both architectural components with batch-adaptive learning, consistently achieves the strongest performance, particularly for rare and clinically critical diseases (Average Rare F1: 0.90). This confirms that effective imbalance mitigation requires a synergistic integration of representation-level and optimization-level strategies rather than isolated improvements.

## 7 Future research directions

While the proposed DAWN-Net framework demonstrates improved robustness under severe class imbalance, several promising research directions remain open. First, extending the current methodology toward *multi-institutional and cross-domain validation* is an important next step. Although large-scale public datasets such as NIH ChestXray14, CheXpert, and PadChest provide diverse training and evaluation settings (28, 29), variations in acquisition protocols, annotation practices, and population demographics continue to pose challenges for generalization. Future studies should therefore investigate domain-adaptive and cross-dataset learning strategies to further improve robustness across heterogeneous clinical environments.

Second, incorporating *uncertainty-aware learning and label ambiguity modeling* represents a valuable extension of the proposed framework. Datasets such as CheXpert explicitly encode uncertainty in radiological labels (28), highlighting the need for models that can reason about ambiguous or weakly supervised annotations. Integrating uncertainty modeling into imbalance-aware optimization may improve reliability in borderline or clinically ambiguous cases, particularly for rare disease categories.

Third, the integration of *explainable artificial intelligence (XAI)* techniques remains an essential direction for clinical adoption. While DAWN-Net improves discriminative performance for underrepresented pathologies, future work should focus on generating clinically interpretable explanations that align model predictions with radiological reasoning. This is especially relevant for rare disease detection, where trust and transparency are critical for clinical decision-making.

Finally, future research may explore the extension of the proposed imbalance-aware learning paradigm to *multi-modal and longitudinal imaging scenarios*. Large-scale datasets with paired imaging and report data, such as those available in de-identified radiology repositories (30), offer opportunities to jointly model visual and textual information. Leveraging such multi-modal cues within an imbalance-aware framework could further enhance diagnostic performance and reliability in real-world radiology workflows.

Overall, these directions highlight the potential for extending DAWN-Net beyond single-modality classification toward more general, interpretable, and clinically deployable AI-assisted medical imaging systems.

## 8 Conclusion

This work addressed a fundamental limitation of AI-assisted chest X-ray classification, namely the persistent degradation in performance for rare but clinically critical diseases caused by severe class imbalance. To overcome this challenge, DAWN-Net was proposed as a novel imbalance-aware deep learning framework that integrates complementary architectural and optimization-level strategies. The Hierarchical Feature Propagation Network enhances sensitivity to subtle and localized pathological patterns, while the Semantic Context Modeling Network enforces global anatomical coherence, together enabling balanced and robust feature representation. In parallel, the batch-adaptive class-penalized learning objective dynamically adjusts optimization behavior to mitigate majority-class dominance without destabilizing training.

Comprehensive experimental evaluation across multiple benchmark datasets demonstrates that DAWN-Net consistently outperforms conventional convolutional baselines and loss-only imbalance mitigation strategies, with particularly pronounced gains for rare disease categories. ROC-based analysis and rare-disease F1-score comparisons further confirm that the proposed framework improves discriminative reliability under long-tailed clinical distributions, rather than merely optimizing aggregate performance metrics dominated by common conditions.

This study highlights the importance of jointly addressing class imbalance at both the representation and optimization levels when designing AI systems for medical imaging. By improving the reliable detection of underrepresented pathologies, DAWN-Net contributes to safer and more reliable AI-assisted radiology workflows. Future work will explore extending the proposed framework to multi-modal imaging, incorporating explainability mechanisms for clinical decision support, and validating performance across broader multi-institutional cohorts.

## Data Availability

The original contributions presented in the study are included in the article/Supplementary material, further inquiries can be directed to the corresponding author.
