# Supplementary material for: Integrating augmentation-aware manifold smoothing and momentum-adjusted loss for handling class imbalance in thoracic disease detection
Source: Front Med (Lausanne). 2026 Jun 17;13:1795508. doi: 10.3389/fmed.2026.1795508 (PMC13318738; doi:10.3389/fmed.2026.1795508)
Supplement: Supplementary file 1 [file Table_1.docx]

# Publicly available dataset used in Experiments presented in the manuscript

1. **NIH ChestXray14 Dataset**: Wang, X., Peng, Y., Lu, L., Lu, Z., Bagheri, M., and Summers, R. M. (2017). ChestX-ray8: Hospital-scale chest x-ray database and benchmarks on weakly-supervised classification and localization of common thorax diseases. In *Proceedings of the IEEE Conference on Computer Vision and Pattern Recognition*, pp. 2097–2106.
2. **CheXpert Dataset**: Irvin, J., Rajpurkar, P., Ko, M., Yu, Y., Ciurea-Ilinca, M., Chute, C., Marklund, H., Haghgoo, B., Ball, R., Shpanskaya, K., et al. (2019). CheXpert: A large chest radiograph dataset with uncertainty labels and expert comparison. *Proceedings of the AAAI Conference on Artificial Intelligence*, 33(01), pp. 590–597. doi:10.1609/aaai.v33i01.3301590.
3. **PadChest Dataset**: Bustos, A., Pertusa, A., Salinas, J.-M., and de la Iglesia-Vayá, M. (2020). PadChest: A large chest x-ray image dataset with multi-label annotated reports. *Medical Image Analysis*, 66, 101797. doi:10.1016/j.media.2020.101797.
